# Case report: Recurrence of inflammatory cardiomyopathy detected by magnetocardiography

**DOI:** 10.3389/fcvm.2023.1225057

**Published:** 2023-09-22

**Authors:** Phillip Suwalski, Ainoosh Golpour, Nicolas Musigk, Finn Wilke, Ulf Landmesser, Bettina Heidecker

**Affiliations:** Department of Cardiology, Angiology and Intensive Care Medicine CBF, Deutsches Herzzentrum der Charité – Universitätsmedizin Berlin, Corporate Member of Freie Universität Berlin and Humboldt – Universität zu Berlin, Berlin, Germany

**Keywords:** inflammatory cardiomyopathy, magnetocardiography, diagnostic method, therapy monitoring, case report

## Abstract

**Background:**

The diagnosis of inflammatory cardiomyopathies remains challenging. Life-threatening conditions such as acute coronary syndrome (ACS) always have to be considered as differential diagnoses due to similarities in presentation. Diagnostic methods for inflammatory cardiomyopathy include endomyocardial biopsy (EMB), cardiac magnetic resonance imaging (CMR), and positron emission tomography-computed tomography (PET-CT). We report a case in whom magnetocardiography (MCG) led to an initial diagnosis of inflammatory cardiomyopathy and in whom MCG was used for subsequent monitoring of treatment response under immunosuppression.

**Case presentation:**

A 53-year-old man presented with two recurrent episodes of inflammatory cardiomyopathy within a 2-year period. The patient initially presented with reduced exercise capacity. Echocardiography revealed a moderately reduced left ventricular ejection fraction (LVEF 40%). Coronary angiography ruled out obstructive coronary artery disease (CAD) and an EMB was performed. The EMB revealed inflammatory cardiomyopathy without viral pathogens or replication. Moreover, we performed MCG, which confirmed a pathological Tbeg-Tmax vector of 0.108. We recently established a cutoff value of Tbeg-Tmax of 0.051 or greater for the diagnosis of inflammatory cardiomyopathy. Immunosuppressive therapy with prednisolone was initiated, resulting in clinical improvement and an LVEF increase from 40% to 45% within 1 month. Furthermore, the MCG vector improved to 0.036, which is considered normal based on our previous findings. The patient remained clinically stable for 23 months. During a routine follow-up, MCG revealed an abnormal Tbeg-Tmax vector of 0.069. The patient underwent additional testing including routine laboratory values, echocardiography (LVEF 35%), and PET-CT. PET-CT revealed increased metabolism in the myocardium—primarily in the lateral wall. Therapy with prednisolone and azathioprine was initiated and MCG was used to monitor the effect of immunosuppressive therapy.

**Conclusion:**

In addition to diagnostic screening, MCG has the potential to become a valuable method for surveillance monitoring of patients who have completed treatment for inflammatory cardiomyopathy. Furthermore, it could be used for treatment monitoring. While changes in the magnetic vector of the heart are not specific to inflammatory cardiomyopathy, as they may also occur in other types of cardiomyopathies, MCG offers a tool of broad and efficient diagnostic screening for cardiac pathologies without side effects.

## Background

Inflammatory cardiomyopathy commonly affects the left ventricle (LV), which may lead to impaired LV function (1). Patients may present with symptoms of heart failure such as reduced exercise tolerance, dyspnea, and fatigue, as well as palpitations and chest pain (2). While the clinical presentation of inflammatory cardiomyopathy is mild in the majority of patients, in some it may present with severe arrhythmias and sudden cardiac death.

Treatment consists of standard heart failure therapy and in specific cases, additional immunosuppressive treatment to reduce myocardial damage, remodeling, and cardiovascular complications. The choice of immunosuppressive agent depends on the etiology (3).

Numerous etiologies of inflammatory cardiomyopathy have been described, including viral (4), bacterial, autoreactive, and autoimmune (5–7). Endomyocardial biopsy (EMB) remains the gold standard for diagnostic confirmation of inflammatory cardiomyopathy. Cardiac magnetic resonance imaging (CMR) is the non-invasive gold standard, which may also be used to identify affected myocardial regions for EMB (8). The availability of both methods is limited due to infrastructure and expertise concerns across medical institutions.

In this case, we present magnetocardiography (MCG) as a potential future complementary diagnostic screening and monitoring tool in inflammatory cardiomyopathy (9, 10). The examination is carried out in a standardized manner that requires 60 s. One of the limitations of MCG is that data from patients with any type of metal in their chest, such as cardiac devices, cannot be interpreted due to possible interference by such devices with the heart's magnetic field.

There are no known side effects since the method is based on sensing the existing magnetic field of the heart without sending out any type of energy itself, e.g., radiation, a magnetic field, or ultrasonic waves.

## Methods: magnetocardiographic measurements

The movement of ions in and out of cardiomyocytes is the basis for the generation of the action potential. Due to ion shifts, voltages are created and thus also an electromagnetic field. The vector of the magnetic field and its strength are affected by the magnitude of intra- and extracellular ion flow (9). The electromagnetic field generated by the heart has a field strength of between 10^−15^ and 10^−11^ T. This field is the basis for both electrocardiography (ECG) and MCG.

The MCG system is an arrangement of 64 highly sensitive magnetic sensors called superconducting quantum interference sensors (SQUIDs) (9). The measuring unit is located in a shielded room to reduce measurement interference from other electromagnetic signals. SQUIDs measure the changes in the magnetic field of the heart during the cardiac cycle and display those changes in accordance with the QRS complex (9). Various frequency filters help to eliminate electromagnetic interference signals. The measurement of the magnetic field has a three-dimensional resolution, so that it is possible to create a sum vector as the main electrical axis of the heart. When diagnosing inflammatory cardiomyopathies, the vector of the action potential T-wave and the T-wave maximum is analyzed (Tbeg-Tmax interval). A vector area of the T-wave/MCG vector Tbeg-Tmax greater than 0.051 is considered pathological (9). A limitation of the MCG Tbeg-Tmax vector is that it is not specific for inflammatory cardiomyopathy. Other types of cardiomyopathy, such as CAD or amyloidosis, may also impact the electromagnetic axis (9, 11).

## Case description

We report a 53-year-old patient who presented with a decrease in exercise tolerance. His past medical history included atrial fibrillation, intermittent left bundle branch block, arterial hypertension, hypercholesterolemia, and seizure disorder. The patient was treated with acetylsalicylic acid, metoprolol succinate, eplerenone, sacubitril/valsartan, ezetimibe, and oxcarbazepine. The family history was remarkable for idiopathic dilated cardiomyopathy. His brother required a heart transplant at the age of 52.

**Figure 1:**
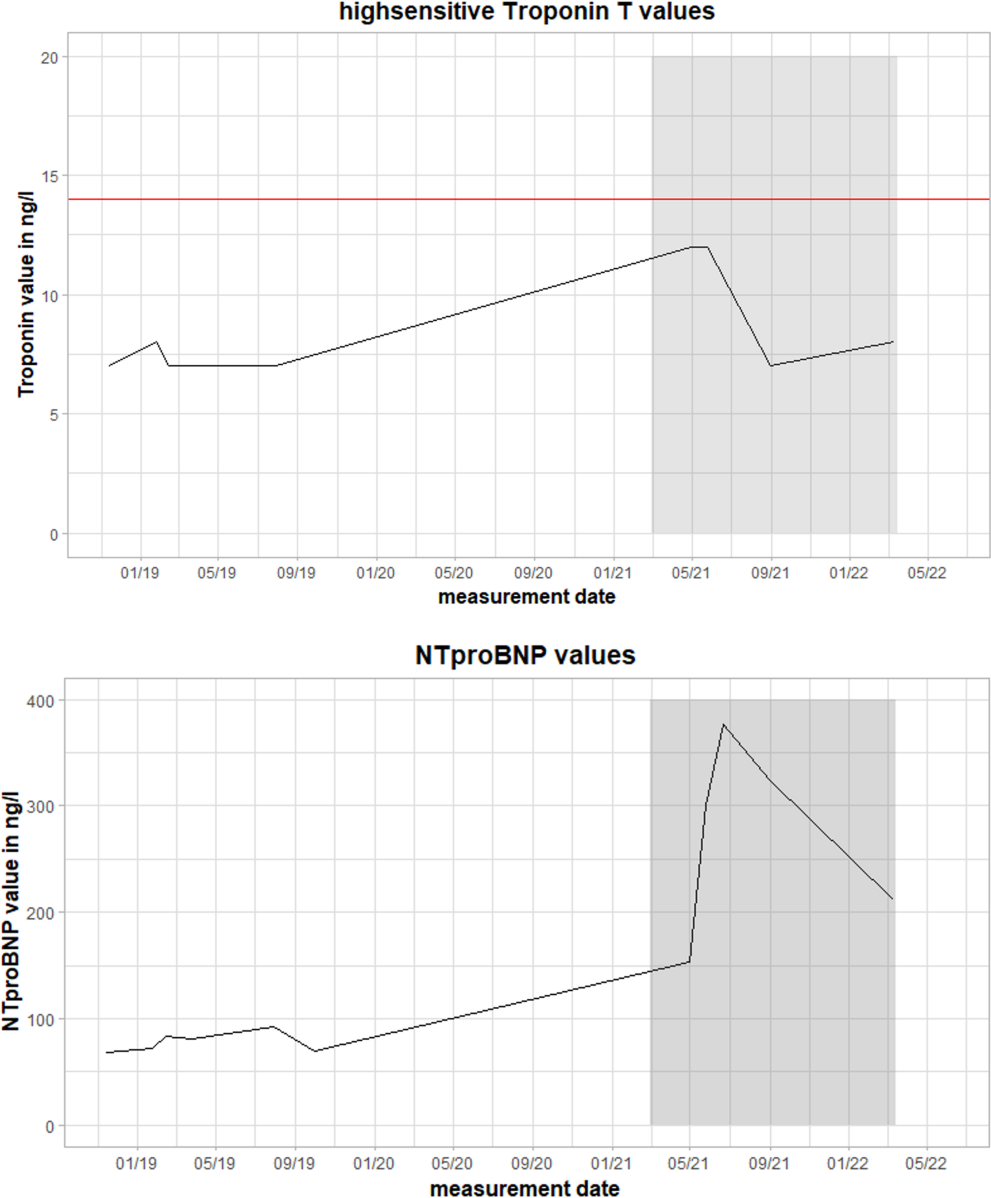
Values of high-sensitivity troponin T (hsTnT) and NT pro brain natriuretic peptide (NTproBNP) during the clinical course. The red line represents the pathological threshold of hsTnT (>14 ng/L). The grey box marks the time between the first and follow-up fluorodeoxyglucose positron emission tomography-computed tomography (FDG-PET-CT) scan during the second episode of myocarditis. Magnetocardiographic measurements took place in this timeframe.

The clinical examination was unremarkable. The patient was hemodynamically stable and euvolemic. There were no murmurs, rubs, or gallops on cardiac auscultation. An electrocardiogram (ECG) revealed sinus rhythm with T-wave inversions in aVL and V4–V6. Holter monitoring ruled out any relevant arrhythmias. On echocardiography, left ventricular ejection fraction was moderately reduced (LVEF 40%) with left ventricular apical and inferior hypokinesis. Relevant results from laboratory testing are illustrated in Figure 1. High-sensitivity Troponin T (hsTnT), NT pro brain natriuretic peptide (NTproBNP), creatine kinase (CK), and CK-MB were not increased. There were no abnormalities suggesting inflammation or infection. C-reactive protein (CRP), procalcitonin (PCT), and differential blood count were within normal ranges.

**Figure 2:**
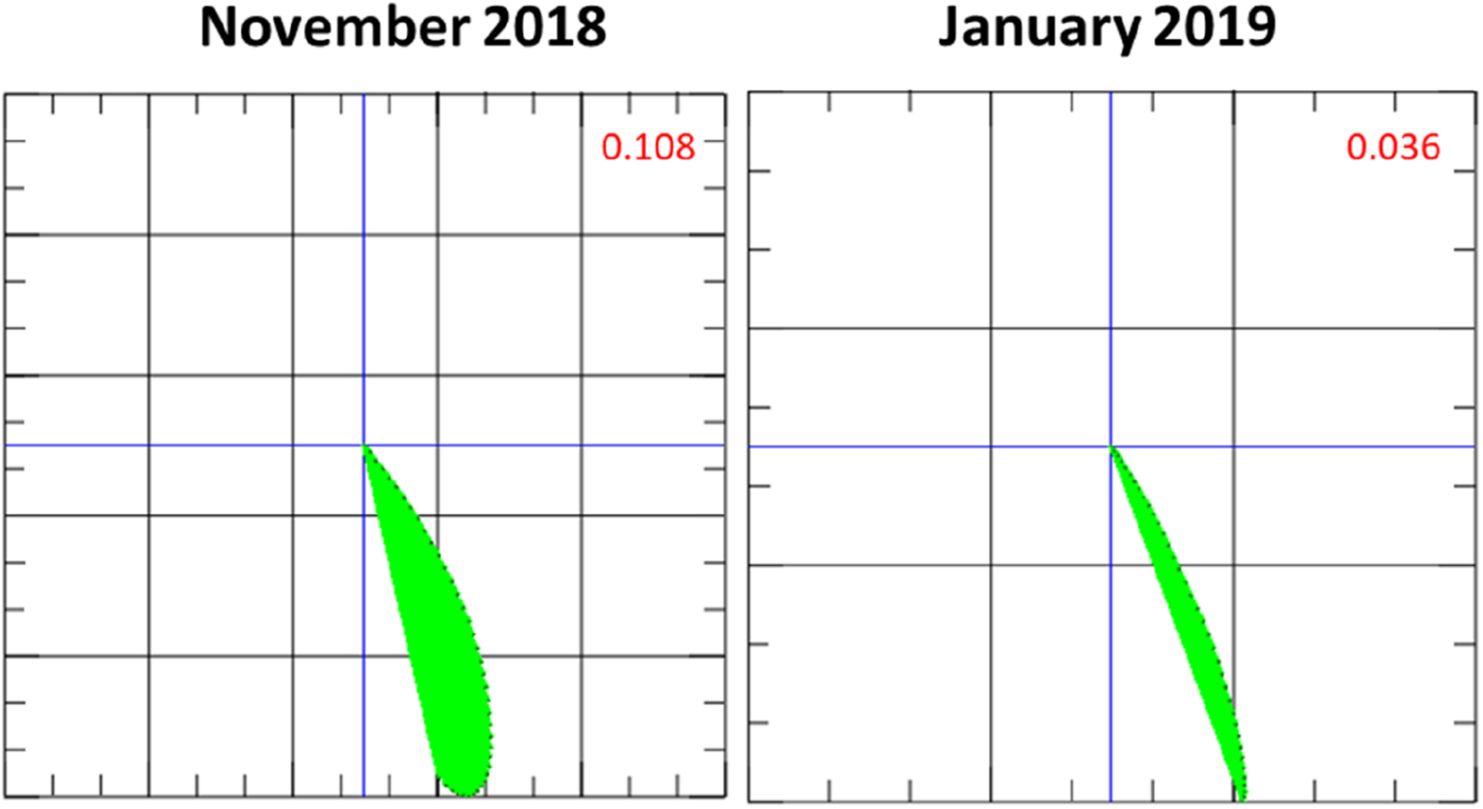
Initial magnetocardiography (MCG) vector and MCG vector after 1 month of immunosuppressive therapy with prednisolone. The MCG vector (green plane) decreases, moving towards a score that is considered normal (<0.051) based on our previous findings (9) and suggestive of a response to therapy.

Coronary artery disease was ruled out with coronary angiography. An EMB was performed, revealing inflammatory cardiomyopathy with increased lymphocytes, macrophages, and increased ICAM-1 expression. Viral pathogens (adenovirus, human herpes virus 6, erythrovirus Z, Epstein–Barr virus, enterovirus) could not be detected. Initial MCG measurement revealed a pathological magnetic Tbeg-Tmax vector of 0.108 [previously established cutoff value for normal Tbeg-Tmax vector <0.051 (9); Figure 2].

After a negative QuantiFERON-test and chest x-ray to rule out active infection with tuberculosis, immunosuppressive therapy was initiated with prednisolone 60 mg daily for 4 weeks followed by a reduction of 10 mg of the daily dose every 2 weeks.

One month after the start of immunosuppressive therapy, LVEF improved to 45%. 12-lead ECG had normalized. An MCG follow-up measurement at that time revealed a relevant decrease of the Tbeg-Tmax vector from 0.108 to 0.036 (reference value <0.051, Figure 2), suggesting therapy response (9).

After having completed a 3-month course of prednisolone therapy, the patient was found to have an almost normalized left ventricular function (LVEF of 51%). The clinical symptoms of chest pressure and reduced physical activity had subsided. 12-lead ECG showed no pathological findings.

During a routine follow-up examination approximately 2 years after the end of the immunosuppression, the patient's left ventricular function had again decreased. Repeat echocardiographic measurements every 4–6 months until then revealed stable LVEF, while at 2-year follow-up, the LVEF was 35%. Similar to his initial presentation with inflammatory cardiomyopathy, the patient was in sinus rhythm with T-wave inversions in V2–V5 (12-lead ECG). The patient reported occasional chest pressure, dyspnea, and fatigue in recent weeks. The physical examination remained unremarkable.

**Figure 3:**
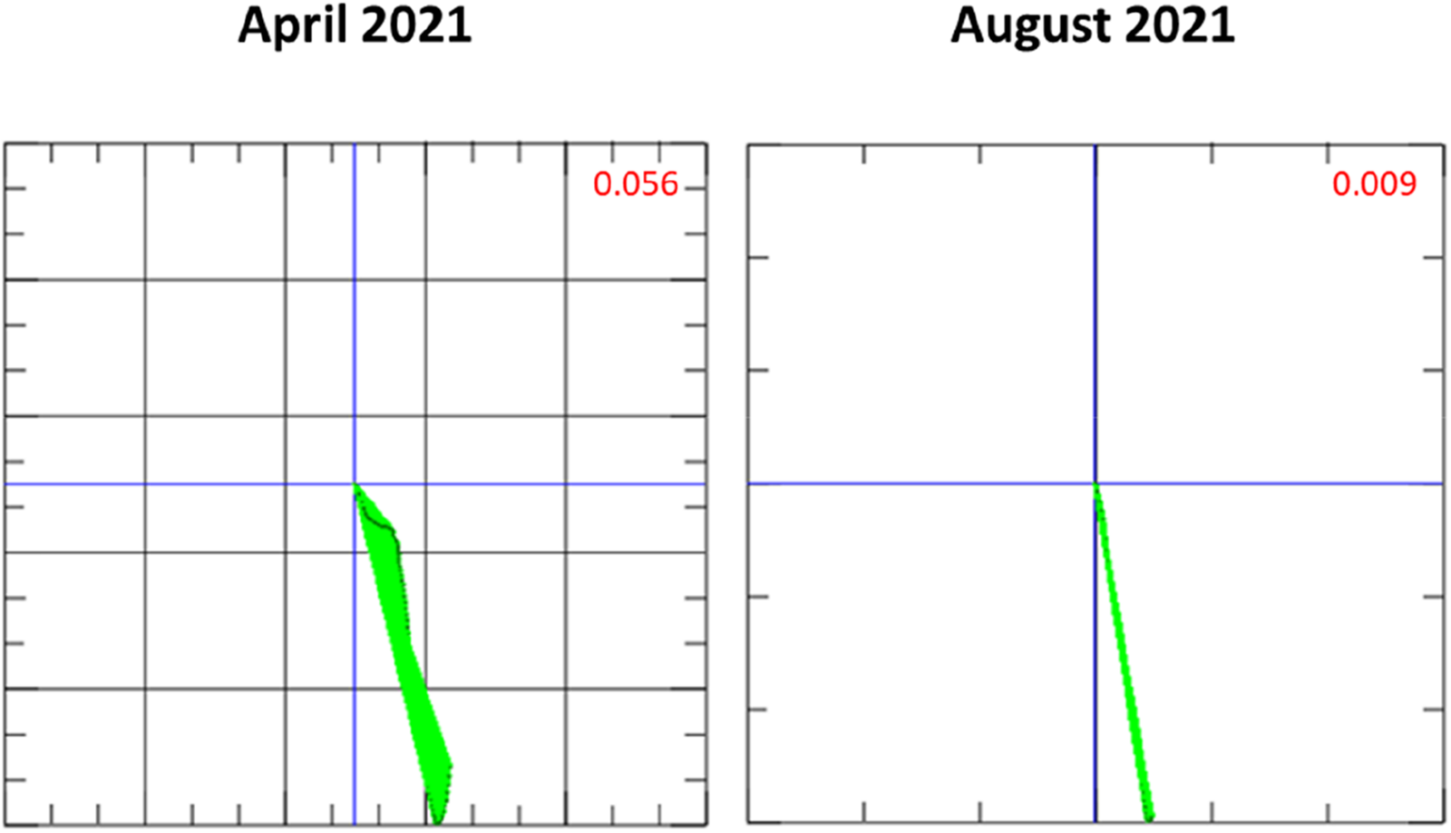
Magnetocardiography (MCG) vector during long-term immunosuppressive treatment throughout the second episode of inflammatory cardiomyopathy. In the time between April and August 2021, the vector normalized (<0.051 is considered normal) (9).

**Figure 4:**
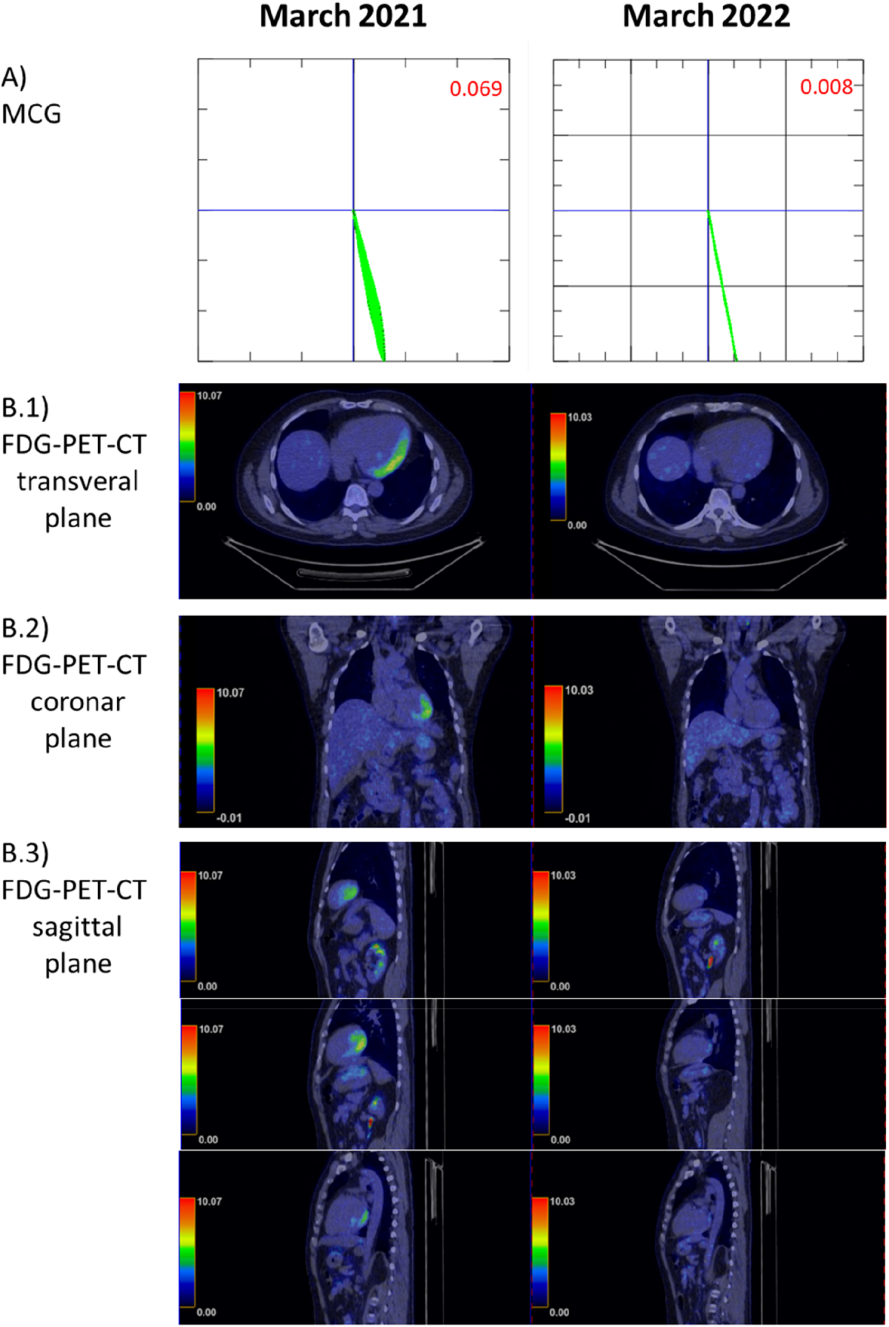
Comparison of fluorodeoxyglucose positron emission tomography-computed tomography (FDG-PET-CT) to the magnetocardiography (MCG) vector before long-term immunosuppressive treatment (March 2021) and afterward (March 2022, 1 year later). (**A**) MCG vector: vector normalization within 1 year (<0.051 is considered normal) (9). (**B**) (**B.1–B.3**) Illustrate the different planes of the FDG-PET-CT and cardiac tracer uptake.

The patient underwent MCG measurement as part of his follow-up examination (Figures 3, 4). MCG supported the recurrence of inflammatory cardiomyopathy by detecting a pathological Tbeg-Tmax vector with a value of 0.069 (reference Tbeg-Tmax <0.051, Figure 4) (9). Fluorodeoxyglucose positron emission tomography-computed tomography (FDG-PET-CT) was used to screen for recurrent inflammation. The FDG-PET-CT revealed an apical, mid-posterior-lateral tracer accumulation, primarily lateral, suggestive of recurrent inflammation. Given these findings, immunosuppressive therapy was initiated with prednisolone 60 mg daily followed by a reduction of the daily dose by 10 mg every 2 weeks. The patient was followed up with clinical examinations and MCG measurements biweekly.

Four weeks after the start of immunosuppressive therapy, there was no significant improvement in the LVEF 30%. MCG measurement revealed a response to prednisolone therapy, with an improved, but still pathological, Tbeg-Tmax value of 0.056 (Figure 3, April 2021) (9). 12-lead ECG had normalized and remained without any pathological findings during the course of the prednisolone treatment.

Six weeks after the start of prednisolone treatment, LVEF was 34%. Coronary angiography was performed for further diagnostic workup. No obstructive coronary artery disease was detected. LVEF remained at 34% on echocardiography. After completion of the prednisolone therapy, a maintenance dose of prednisolone 5 mg daily for at least 1 year was recommended. An MCG measurement in August 2021 presented a significant improvement in the Tbeg-Tmax vector, with a value of 0.009 recorded. An FDG-PET-CT scan at 1-year follow-up (after the recurrence of myocarditis) was performed. Compared to March 2021, there was a significant reduction of tracer uptake in previously active areas. Minimal residual tracer accumulation was still present in the posterior left ventricle (Figures 4B1, B3).

Given the persistent minimal residual inflammatory activity, the patient was treated with azathioprine 2 mg/kg body weight and prednisolone 5 mg over the course of 6 months. At 6 months follow-up, the patient continued to be clinically stable and reported no cardiac symptoms, e.g., dyspnea or chest pressure. The LVEF remained at 34%.

## Discussion

In this case report, we present a 53-year-old male patient suffering from recurrent inflammatory cardiomyopathy of unknown etiology. FDG-PET-CT, MCG, and left-ventricular EMB were used for diagnosis. Furthermore, MCG was used as a supportive therapy monitoring method. This case demonstrates the importance of considering inflammatory cardiomyopathy even in cases with minimal symptoms on presentation. Cardiac-specific laboratory parameters such as NTproBNP, hsTnT, and CK-MB were within normal ranges when the patient initially presented and during his follow-up visits. The combination of echocardiography, ECG, FDG-PET-CT, EMB, and MCG led to a diagnosis of inflammatory cardiomyopathy.

Chow et al. (12) reported in their study that EMB with 4–5 samples/patient could correctly identify inflammatory cardiomyopathy in 43%–57% of patients. However, the sensitivity varies with the number of biopsy samples (13). The detection rate may be increased up to 89% using guidance methods for EMB (a combination of CMR and electroanatomic voltage mapping) (14, 15). Imaging methods such as CMR or FDG-PET-CT have been reported to have higher detection rates—69% with CMR and 74% with FDG-PET-CT—but EMB is needed for definitive subtyping of the diagnosis and detection of the etiology (16, 17). For monitoring of therapy response, regular use of EMB, CMR, or FDG-PET-CT is not feasible in small intervals, given the procedural risk, limited availability, and the risk of radiation exposure. Clinical course, laboratory values (e.g., hsTnT or NTproBNP, ECG), and echocardiography are typically used for monitoring of treatment response. However, their sensitivity for the detection of inflammatory activity is limited (18). Follow-up is particularly challenging in patients with an oligo- or asymptomatic clinical course and normal laboratory values.

Overall, it has been suggested that LVEF is generally not a reliable parameter for monitoring the course of inflammatory cardiomyopathies (19) because the inflammatory activity is not directly linked to the LVEF. Structural changes such as myocardial scars and fibrosis can lead to a permanent decrease in LVEF, which may not necessarily indicate ongoing inflammation. This may be a possible explanation why the LVEF did not improve in this case. ECG is also limited, as the presented case shows. The 12-lead ECG normalized, despite the presence of symptoms and still persistent inflammation. MCG offers new possibilities for therapy monitoring since measurements take approximately 1 min and there are no side effects.

Changes in the magnetic field of the heart are not specific to a certain cardiac disease. Nevertheless, MCG is a valuable tool to screen for inflammatory cardiomyopathy in particular scenarios in which the pretest probability for CAD is low or CAD has been excluded. Furthermore, the MCG Tbeg-Tmax vector may represent an objective parameter to evaluate the response to immunosuppressants. The present patient had an initial MCG Tbeg-Tmax vector of 0.108. Four weeks after the start of prednisolone therapy, the Tbeg-Tmax vector improved to 0.036. The follow-up measurements revealed a decreasing MCG Tbeg-Tmax vector, consistent with a response to prednisolone therapy (Figures 2–4), which agrees with the improvement of initial dyspnea and chest pain symptoms. This improvement was confirmed at 1-year follow-up with FDG-PET-CT.

The limitations of MCG become apparent when comparing the MCG vector from 2022 with its corresponding FDG-PET-CT. Very weak residual inflammatory activity in this treated patient was not detected as a pathological MCG vector. While we see continuous improvement of the MCG vector during therapy response, it remains to be evaluated if a specific vector change (ΔMCG) indicates that inflammation is fully suppressed or if a final absolute MCG vector value has to be achieved to consider a therapy successfully completed. MCG is very unlikely to replace any established diagnostic tools in inflammatory cardiomyopathy, but our data suggest that it is a valuable screening tool for diagnosis, monitoring of treatment response, and long-term surveillance in inflammatory cardiomyopathy, given its non-invasiveness, practicality, and the absence of side effects and radiation. Furthermore, the described Tbeg-Tmax vector is only one parameter acquired during the MCG measurements. Other parameters such as magnetic field distribution maps and current density maps are still under evaluation for diagnosing and monitoring patients with inflammatory cardiomyopathy.

## Conclusion

MCG has potential as a novel diagnostic and monitoring tool for patients with inflammatory cardiomyopathies, offering advantages such as minimal side effects, short measurement time, and the potential for obtaining objective clinical parameters for clinical decision making. A limitation of the MCG Tbeg-Tmax vector is that it is not specific for inflammatory cardiomyopathy. Other types of cardiomyopathy such as CAD or amyloidosis may also impact the electromagnetic axis (9, 11). MCG is currently (April 2023) used only for research purposes. Further studies are necessary to evaluate its value for broad clinical applications.

## Data Availability

Data from the study can be made available upon request from the principal investigator Bettina Heidecker in a de-identified form. A data use agreement must be signed before. Access can only be granted to academic staff.
